# Propionate-engineered probiotics reduce radiation-induced intestinal damage

**DOI:** 10.1186/s40643-026-01020-9

**Published:** 2026-02-17

**Authors:** Xinran Gao, Jiahao Wu, Kaihua Ji, Mengxue Gao, Yufei Guo, Lina Wang, Xiaoxiao Jia, Xinran Lu, Zhixin Zhu, Qinghua Wang, Ping Wang, Zhenyu Zhao, Guangbo Kang, Qiang Liu, He Huang

**Affiliations:** 1 School of Synthetic Biology and Biomanufacturing, State Key Laboratory of Synthetic Biology, Tianjin Key Laboratory of Biological and Pharmaceutical Engineering, Tianjin University, Tianjin, 300350 China; 2 Institute of Radiation Medicine, Tianjin Key Laboratory of Radiation Medicine and Molecular Nuclear Medicine, State Key Laboratory of Advanced Medical Materials and Devices, Chinese Academy of Medical Sciences & Peking Union Medical College, Tianjin Institutes of Health Science, Tianjin, 300192 China; 3 Department of Anatomy, Shandong Second Medical Universtiy, Weifang, 261053 China; 4 Tianjin Modern Innovative TCM Technology Co. Ltd, Tianjin, 300392 China; 5 NHC Key Laboratory of Hormones and Development, Tianjin Institute of Endocrinology, Chu Hsien-I Memorial Hospital, Tianjin Medical University, Tianjin, 300134 China; 6 School of Population Medicine and Public Health, Chinese Academy of Medical Sciences & Peking Union Medical College, Beijing, 100730 China; 7 Haihe Laboratory of Sustainable Chemical Transformations, Tianjin, 300192 China

**Keywords:** Radiation, Propionate synthetic biology, Engineered probiotics, Gut, Gut microbiota

## Abstract

Radiation enteritis is a common complication in patients undergoing abdominal radiotherapy. Current management strategies face significant limitations: clinical agents like amifostine are hindered by systemic side effects and demanding administration; direct supplementation with radioprotective metabolites such as propionate suffers from low bioavailability and transient action; and conventional probiotics lack targeted therapeutic output. To address these challenges, we engineered *Escherichia coli* Nissle 1917 to function as a living therapeutic that continuously produces and delivers propionate directly in the gut. This propionate-engineered probiotic achieved a production yield of 181.33 ± 4.27 mg/L in vitro. In a mouse model of abdominal irradiation, this engineered bacterium alleviated radiation-induced intestinal damage by continuously releasing propionate and enhancing intestinal epithelial barrier function. Multi-omics analysis revealed that the engineered bacterium could restore intestinal microbiota homeostasis, enhancing the abundance of advantageous bacteria with radioprotective properties (e.g., *Dubosiella*, *Akkermansia*). Moreover, it modulated intestinal microbiota metabolism, influencing the metabolism of ascorbic acid, aldoses, and other metabolites. Additionally, it protected the intestinal mucosal barrier from radiation-induced damage, which was associated with the modulation of the SOCS1/JAK2/STAT3 signaling pathway. This study introduces a novel biological therapy to mitigate the side effects of radiotherapy and could open new avenues for preventing and treating radiation-induced intestinal injury.

**Supplementary Information:**

The online version contains supplementary material available at 10.1186/s40643-026-01020-9.

## 1. Introduction

Radiation therapy, which utilizes high-energy rays (e.g., X-rays, γ-rays, high-energy electrons, or heavy particles), eradicates cancer cells by inducing DNA damage. It can be administered independently or in combination with surgery or chemotherapy (Abdel-Wahab et al. 2024). Although ionizing radiation effectively targets tumor cells, it also damages healthy tissues, including the intestinal mucosa, nerves, and vasculature. This damage leads to a range of gastrointestinal complications, from acute symptoms like abdominal pain, diarrhea, and vomiting to chronic conditions such as intestinal stenosis, obstruction, hemorrhage, and fistulas (Giridhar et al. 2024). Radiation enteritis is a frequent complication in patients receiving abdominal radiotherapy, with an incidence of acute enteritis ranging from 50% to 70%(Jian et al. 2021). Abdominal irradiation can cause inflammation, increase intestinal barrier permeability, and damage the intestinal mucosa (Andreyev et al. 2010).

Current clinical treatments for radiation enteritis are limited and primarily focus on symptomatic relief, including anti-infection therapy, nutritional support, and antidiarrheal measures (Cerecedo et al. 2023). Unfortunately, no specific drug has been internationally approved for its prevention or cure (Wang et al. 2025). Octreotide, which suppresses gastrointestinal secretions and alleviates diarrhea, is limited by its high cost and endocrine-disruptive effects during prolonged use (Ma et al. 2019; Zhao et al. 2025). Amifostine, a cytoprotective agent approved by the U.S. Food and Drug Administration (FDA), possesses the ability to scavenge reactive oxygen species (ROS) and is widely utilized for the prevention and treatment of radiation sickness (Hauer-Jensen et al. 2014). However, its administration requires rapid intravenous infusion and is associated with severe adverse effects, such as hypotension (Ruysscher et al. 2019; King et al. 2020). Although existing therapies can alleviate some symptoms, they are still limited by factors such as high cost, insufficient targeting, and susceptibility to adverse effects, restricting their clinical application.

Short-chain fatty acids (SCFAs), key metabolites produced by the gut microbiota, are essential for maintaining intestinal homeostasis (Mann et al. 2024; Yang et al. 2020). Among these, butyrate has received significant attention in intestinal injury research (Eshleman et al. 2024; Yang et al. 2024a, b). However, studies indicate that propionate, more than acetate or butyrate, enhances survival in irradiated mice and exhibits stronger radioprotective effects (Guo et al. 2020; He et al. 2023). Propionate protects the intestinal mucosal barrier from radiation-induced damage by upregulating tight junction proteins and mucin-2^17^, and promotes epithelial renewal and repair by increasing cellular motility and persistence (Bilotta et al. 2021). Additionally, propionate supports metabolic processes (Yao et al. 2022), stimulates intestinal tissue development, and strengthens immune function (Liu et al. 2023), showing therapeutic potential in treating inflammatory bowel disease (IBD) (Ma et al. 2020; Peng et al. 2022; Shin et al. 2023). However, the direct oral supplementation of propionate is limited by low bioavailability and transient effects (Zhang et al. 2020), due to its rapid proximal absorption and non-physiological pulsatile exposure, which may not adequately mimic the continuous, local production critical for its optimal function (Schmitt et al. 1976, 1977; Binder et al. 2014; Guan et al. 2021; Ye et al. 2025; Kang et al. 2024). This highlights the need for novel biotherapeutic strategies capable of providing sustained, in situ delivery of propionate within the intestinal niche.

Probiotics, beneficial microorganisms that support gut health, are vital for maintaining intestinal homeostasis and enhancing immunity (Xia et al. 2024). With advancements in synthetic biology, probiotics have emerged as excellent vectors for the targeted delivery of bioactive substances(Heavey et al. 2024). Research has shown that *Escherichia coli* Nissle 1917 (EcN), with its superior colonization capacity and genetic stability, is an ideal chassis organism (Redenti et al. 2024; Gurbatri et al. 2024). Moreover, as a probiotic, EcN promotes intestinal tissue repair and functional recovery through mechanisms such as protecting the intestinal barrier, modulating immunity, and scavenging reactive oxygen species (ROS) (Zhou et al. 2022; Hu et al. 2024). Its synergistic action with propionate further enhances therapeutic outcomes. Consequently, EcN has been engineered as a vector for the targeted release of propionate. Genetic engineering of probiotics to produce propionate not only overcomes the limitations of direct supplementation but also harnesses the colonization and proliferation traits of probiotics to provide sustained propionate delivery, thereby amplifying its beneficial effects on intestinal health. Building on this foundation, our propionate-producing engineered probiotic represents a convergent strategy designed to overcome the collective limitations of existing options. Unlike systemic pharmacologics, it minimizes off-target effects through gut-restricted action; unlike direct metabolite supplementation, it enables sustained, localized delivery; and unlike conventional probiotics, it provides targeted, high-level production of a specific therapeutic agent (propionate). This integrative approach combines the sustained therapeutic action of propionate with the multifunctional benefits (e.g., barrier reinforcement, immunomodulation) of a probiotic chassis within a single, colonizing entity.

**Scheme 1:**
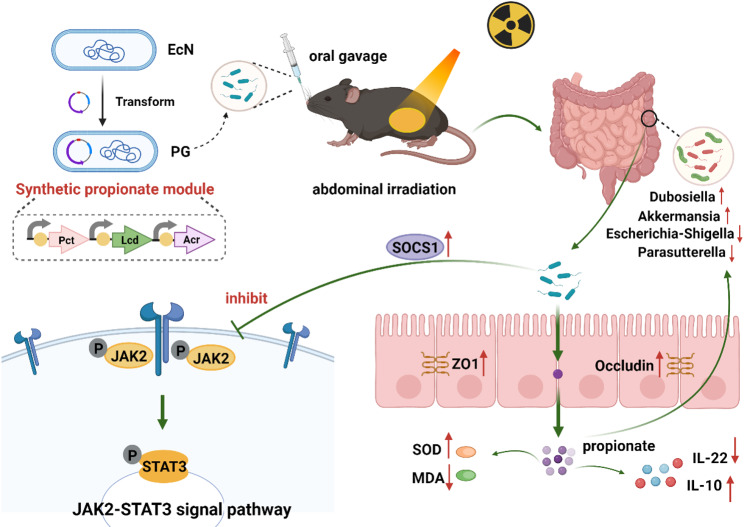
Schematic illustration of the PG therapeutic

Therefore, we developed a series of propionate-producing engineered probiotics based on this rationale. To assess whether these engineered probiotics could alleviate radiation-induced intestinal injury, we evaluated their ameliorative effects using an abdominal irradiation injury model. The results demonstrated that the propionate-producing engineered probiotics could stably and efficiently release propionic acid in the intestinal tract, providing significant radioprotection to the small intestine. Furthermore, 16 S rRNA gene sequencing and metabolomics analysis revealed the effects of these probiotics on intestinal microbiota and metabolism. The engineered probiotics were found to regulate microbiota homeostasis by enhancing the relative abundance of radioprotective and therapeutic strains while decreasing harmful bacteria. Additionally, they modulated microbial metabolism, enhancing beneficial metabolite levels and reinforcing related metabolic pathways, which helped protect against radiation-induced intestinal damage. The engineered probiotics can also strengthen the intestinal barrier and promote intestinal health by regulating signaling pathway. This study introduces a novel biological therapy for alleviating radiation-induced intestinal injury, and propionate-producing engineered probiotics show promise as a clinical approach for prevention and treatment, potentially improving patients’ quality of life Scheme 1

## 2. Materials and methods

### 2.1 strains and culture conditions

*E. coli* Nissle 1917 (EcN) was stored in our laboratory. *Escherichia coli* DH5α was purchased from Transgen Biotech, China. EcN and *E. coli* DH5α were cultured in Luria-Bertani (LB) broth or grown on LB agar plates containing streptomycin (50 µg/mL), chloramphenicol (50 µg/mL), and ampicillin (100 µg/mL) at 37 °C.

### 2.2 construction of propionate-producing engineered probiotics

Full strain and plasmid information is presented in Supplementary Table S1. The propionate coenzyme A-transferase gene *Pct* (GenBank accession number: AJ276553), the lactoyl-CoA dehydratase gene *Lcd* (GenBank accession number: JN244651-3), and the acryloyl-CoA reductase gene *Acr* (GenBank accession number: JN244654-6) were derived from *Clostridium propionicum* DSM 1682. The genes were codon-optimized for *E. coli*, synthesized, and assembled into a recombinant expression vector. Briefly, the native IPTG-inducible promoter of the pCDFDuet-1 plasmid was replaced with the constitutive promoter J23105 via restriction digestion and ligation. The Pct, Lcd, and Acr genes were then sequentially cloned into the multiple cloning sites of the modified vector. This generated the final plasmid, designated pCDFDuet-Pct-Lcd-Acr. The plasmid was verified by diagnostic restriction digestion and Sanger sequencing. Agarose gel electrophoresis confirming the correct size of the purified plasmid and key restriction fragments is provided in Supplementary Figure S1. This recombinant plasmid was subsequently transformed into electrocompetent EcN cells via electroporation to construct the engineered strain PE.

### 2.3 bypass gene knockout via homologous recombination

Genes involved in acetic acid synthesis and metabolism were knocked out using homologous recombination. In the first step, knockout fragments were generated by amplifying the invertase recognition site FRT, along with the resistance gene expression element flanked by FRT sites, using pKD3 as a template. PCR was then used to ligate the FRT and resistance gene expression element, resulting in the construction of the knockout fragment, which consists of the upstream homology arm, FRT, resistance gene expression element, FRT, and the downstream homology arm. In the second step, the strain to be knocked out, containing the pKD46 plasmid, was prepared, followed by electrotransformation to introduce the knockout fragment into the strain. The third step involved preparing the strain in a receptive state while maintaining the pKD46 plasmid. In the fourth step, the knockout fragment, consisting of the upstream homology arm + FRT + resistance gene expression element + FRT + downstream homology arm, was introduced into the strain via electrotransformation. After comparing the sizes of the PCR products from the knockout strain, the PCR products were sent for sequencing. In the fifth step, the resistance gene in the knockout strain was eliminated.

### 2.4 analysis of propionate content

Gas chromatography-mass spectrometry (GC-MS) was used to determine propionate in the fermentation broth. The sample pretreatment procedure was as follows: the fermentation broth was centrifuged at 12,000 rpm for 2 min, and 5 mL of the supernatant was extracted. Then, 1 mL of 50% H₂SO₄ and 2 mL of ethyl ether were added to the supernatant, which was shaken for 50 min. Subsequently, anhydrous calcium chloride was added to dehydrate the solution, which was then filtered using a 0.22 μm membrane before analysis. The analytes were detected using an Agilent GC-Triple quadrupole mass spectrometer with an injection volume of 1 µL and a flow rate of 1 mL/min. The injection probe was cleaned twice, first with ether and then with dichloromethane. The mass spectrometry conditions were as described in the literature (Kang et al. 2024).

### 2.5 animals

The C57BL/6J mouse strain was selected for this study as it represents a widely used and well-established model in radiation biology, particularly for radiation-induced intestinal injury. Its prevalent application ensures model consistency, reproducibility, and facilitates direct comparison with a substantial body of existing literature (Bensemmane et al. 2021; Cui et al. 2017; Feng et al. 2023; Kwak et al. 2021; Zhou et al. 2024). Accordingly, this study utilized a total of eighty 6–8 week-old male C57BL/6J mice with an initial body weight of 18–20 g, purchased from Beijing HFK Bioscience Co., Ltd. (Beijing, China). Male mice were employed in this preliminary efficacy assessment to minimize the experimental variability associated with hormonal cycles. The mice were housed in groups of five per cage in a specific pathogen-free (SPF) environment at the Institute of Radiation Medicine (IRM), Chinese Academy of Medical Sciences (CAMS). They were maintained under standard conditions with a stable diet and access to sterile water.The experimental protocol received approval from the Institutional Animal Care and Use Committee of the Institute of Radiation Medicine, Chinese Academy of Medical Sciences, and Peking Union Medical College (Approval No. IRM2-IACUC-2411-009). All animal experiments adhered to the National Research Council’s Guide for the Care and Use of Laboratory Animals.

### 2.6 treatment and irradiation process of probiotics EcN and Propionate-Engineered probiotic PG

The mice were randomly assigned to four primary experimental groups: the control group, the radiation-only group (IR), the probiotic chassis intervention group (IR + EcN), and the engineered probiotic intervention group (IR + PG). Except for the control group, the other three groups were further divided into three subgroups based on radiation dose (8 Gy, 12 Gy, and 16 Gy), resulting in a total of 10 subgroups, with each subgroup consisting of 8 mice.The doses of 8, 12, and 16 Gy were selected based on established murine abdominal irradiation models reported in recent literature, where the 8–16 Gy range is commonly used to induce a predictable spectrum of intestinal injuries. For instance, He et al. employed an 8 Gy dose to investigate the role of *Akkermansia muciniphila* in radiation-induced intestinal damage (He et al. 2023), while Zhang et al. used an 11 Gy dose to establish a model for evaluating the radioprotective effect of ferulic acid (Zhang et al. 2024a, b). Furthermore, studies by Lu et al. and Bensemmane et al. have utilized 15–16 Gy doses to model severe acute gastrointestinal syndrome (Bensemmane et al. 2021; Lu et al. 2019). Employing three graded doses within this range allowed for a systematic evaluation of the protective efficacy of our propionate-engineered probiotic against varying injury severities. This design also helped assess potential dose-dependent effects and identify the injury model in which the intervention was most effective, thereby providing a basis for selecting an appropriate dose for subsequent in-depth mechanistic studies. (1) Mice were maintained under standard conditions with a regular diet in the control group. (2) Mice were anesthetized via intraperitoneal injection of 10% chloral hydrate and exposed to abdominal irradiation (ABI) at doses of 8 Gy, 12 Gy, or 16 Gy using the Gammacell 40 Exactor (Atomic Energy of Canada Ltd., Chalk River, Canada) at a rate of 1.0 Gy/min. (3) Mice received a daily oral dose of 200 µL of EcN suspension (10⁹ CFU/mL) for two weeks before irradiation. After the administration period, mice were subjected to 8 Gy, 12 Gy, or 16 Gy ABI. (4) Mice received a daily oral dose of 200 µL of propionate-producing engineered probiotic (PG) suspension (10⁹ CFU/mL) for two weeks before irradiation. They were subsequently exposed to 8 Gy, 12 Gy, or 16 Gy ABI. This radiation protocol, including the equipment, dose rate, and shielding method, is consistent with the established methodology previously employed by our research group for modeling acute radiation enteropathy (Wu et al. 2025).

### 2.7 hematoxylin and Eosin (H&E) staining and immunohistochemistry (IHC) staining

Small intestinal tissues from dissected mice were washed with phosphate-buffered saline (PBS), fixed in 4% formaldehyde, embedded in paraffin, and sectioned into 5 μm thick slices. One set of paraffin-embedded sections was stained with hematoxylin and eosin (H&E). Another set underwent antigen retrieval and was incubated overnight at 4 °C with primary antibodies against Occludin and ZO-1 (Abcam). The sections were then treated with secondary antibodies, followed by color development and microscopic observation.

### 2.8 Inflammation, oxidative stress factor content assay

Measure the levels of the pro-inflammatory factor IL-22 and the anti-inflammatory factor IL-10 in mouse intestinal tissue using ELISA kits (Elabscience, E-EL-M2446 and E-EL-M0046). Assess oxidative stress-related markers MDA and SOD in mouse intestinal tissue with corresponding reagent kits (Elabscience, E-BC-K020-M and E-BC-K025-M).

### 2.9 16 S rRNA sequencing

Genomic DNA was extracted from the contents of the mouse ileum using the Power Fecal DNA Isolation Kit (MoBio, Carlsbad, CA, USA). DNA concentration and purity were assessed by agarose gel electrophoresis and Nanodrop. PCR amplification was conducted using primers specific to the V4 variable region of the 16 S rRNA gene. The PCR products were then purified and ligated with Illumina-compatible adapters to construct sequencing libraries. These libraries were subsequently loaded onto the Illumina HiSeq platform for paired-end sequencing and analysis.

### 2.10 metabolomics analysis

Metabolites in biological samples (e.g., serum, urine, tissues, or cells) are systematically analyzed using liquid chromatography-mass spectrometry (LC-MS) or gas chromatography-mass spectrometry (GC-MS) techniques. First, the samples are extracted with an organic solvent (e.g., methanol or acetonitrile) and then centrifuged to remove proteins and impurities, yielding a supernatant for subsequent analysis. In LC-MS analysis, a reversed-phase column and a gradient elution procedure are employed to separate metabolites, in conjunction with high-resolution mass spectrometry for detection. Scanning modes include both positive and negative ion modes to ensure broader metabolite coverage. After preprocessing the raw data through peak extraction, alignment, and normalization, multivariate statistical analysis is applied to identify differential metabolites. Metabolite databases (e.g., KEGG) are then used for annotation and pathway enrichment analysis, elucidating functional changes in metabolites within biological processes and their correlation with physiological or pathological states. The details are the same as in the previous article (Wu et al. 2025).

### 2.11 transcriptomics analysis

Total RNA was extracted from small intestine tissues using Trizol (Invitrogen, Carlsbad, California, USA), followed by cDNA library construction through fragmentation, reverse transcription, and junction ligation. The libraries underwent quality testing before being sequenced via high-throughput sequencing on the Illumina HiSeq platform. After quality control, the raw data were aligned to the reference genome or transcriptome, and gene expression levels were quantified using specialized software. Based on the quantitative results, differential expression analysis tools were employed to identify differentially expressed genes, and pathway enrichment analyses were conducted using functional annotation databases. Among the differentially expressed genes, those with a fold change greater than 1 and a significance level of *p* ≤ 0.05 were selected.

### 2.12 Western blot analysis

Mouse intestines were lysed in RIPA lysis buffer (containing PMSF and protein phosphatase inhibitors at a ratio of 100:1:1) for 30 min and centrifuged at 12,000 rpm for 10 min at 4 °C to collect the supernatant. Protein concentration was determined using a BCA kit, and protein samples were separated by 10% SDS-PAGE electrophoresis under wet conditions at 90 V for 100 min, then transferred onto a 0.22 μm PVDF membrane. The membranes were blocked with 5% skimmed milk-TBST for 2 h and incubated with primary antibodies at 4 °C overnight. The primary antibodies included anti-SOCS1 (PSH09-68), JAK2 (SY0245), p-JAK2 (SY24-03), STAT3 (SY24-08), p-STAT3 (SZ43-01), and GAPDH (SA30-01), all purchased from HuaBio-Antibodies Co., Ltd (Hangzhou, China). The membranes were washed three times with TBST and incubated with a goat anti-rabbit IgG secondary antibody for 2 h at room temperature. Finally, the target proteins were detected by chemiluminescence.

### 2.13 statistical analysis

GraphPad Prism 9.0.0 software was used for statistical analysis. All data are presented as mean ± SD. An independent sample t-test was used to compare two groups, while one-way ANOVA or two-way ANOVA was performed to analyze differences among multiple groups. Statistical significance was defined as *p* < 0.05. The significance levels were denoted as follows: n.s. (*p* > 0.05), * (*p* < 0.05), ** (*p* < 0.01), *** (*p* < 0.001), and **** (*p* < 0.0001). Results with *p* > 0.05 were considered not significant (n.s.).

## 3. Results

### 3.1 construction of propionate-producing engineered probiotics

**Fig. 1:**
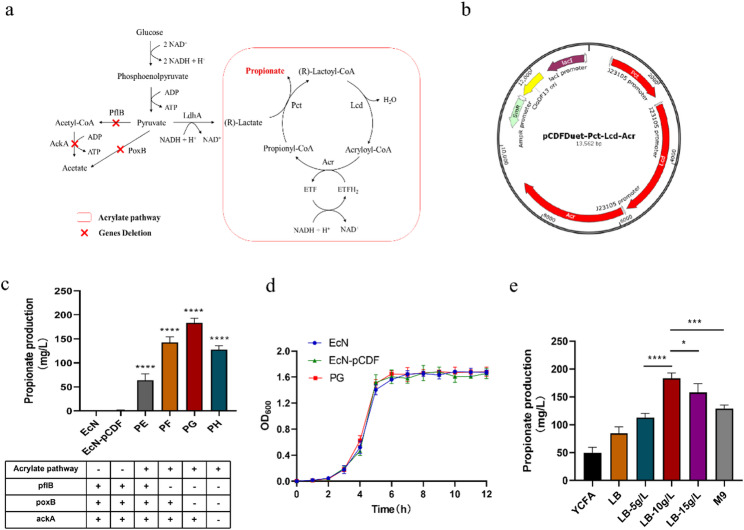
Construction of propionate-producing engineered probiotics. **a** Metabolic pathway of propionate. Genes Pct, Lcd and Acr were introduced. Genes PflB, PoxB and AckA were knocked out. **b** Plasmid mapping of the recombinant plasmid pCDFDuet-Pct-Lcd-Acr. The position and direction of transcription of recombinant genes are indicated by arrows; SmR Streptomycin resistance gene. **c** The amount of propionate produced by EcN, PE, PF, PG and PH. **d** The growth curve of EcN and PG. **e** Propionate production after fermentation of the engineered bacterium PG in different media. LB-5 g/L: LB liquid medium with 5 g/L dextrose; LB-10 g/L: LB liquid medium with 10 g/L dextrose; LB-15 g/L: LB liquid medium with 15 g/L dextrose. Statistical analysis was conducted using one-way ANOVA. Error bars indicate standard errors, and values are expressed as the mean ± SD. *p < 0.05, **p < 0.01, ***p < 0.001, ****p < 0.0001; ns, no significance

To overcome the challenges of low absorption efficiency and limited duration of action associated with oral propionate administration (Shin et al. 2023), we developed an engineered probiotic system using a synthetic biology approach. The goal was to enhance its biosynthetic capacity and achieve sustained release in the digestive tract. We selected EcN as the chassis for the engineered bacteria due to its excellent colonization capacity and genetic stability (Redenti et al. 2024; Gurbatri et al. 2024). EcN, as a probiotic, is capable of metabolically producing butyric acid (BA), acetic acid (AA), and L-lactic acid, but it lacks a metabolic pathway for propanoic acid (PA) synthesis (Miscevic et al. 2020). This is similar to most *E. coli* strains, which naturally produce neither propionate nor its precursor, propionyl-CoA. To address this, we engineered a strain (PE) by using EcN as the chassis and introducing the propionate synthesis pathway-the acrylic acid pathway (Fig. 1a). The plasmid pCDFDuet-Pct-Lcd-Acr, containing the genes for propionate coenzyme A-transferase (*pct*), lactoyl-CoA dehydratase (*lcd*), and acryloyl-CoA reductase (*acr*), was transformed into EcN, regulated by promoter J23105 (Fig. 1b). The supernatant of PE was analyzed by GC-MS, and the results indicated that the recombinant strain PE produced 61.78 ± 3.45 mg/L of propionate (Fig. 1c).

According to the primary metabolic pathway of SCFAs, AA is the major branch of propionate metabolism, influencing the synthetic pathway of propionate (Li et al. 2017). Therefore, knocking down genes related to AA anabolism is one strategy to improve propionate synthesis. Using homologous recombination, we knocked out the *pflB*, *poxB*, and *ackA* genes involved in AA synthesis in the PE genome, generating strains PF, PG, and PH. Among these, the simultaneous knockout of *pflB* and *poxB* effectively reduced AA production and increased propionate synthesis (PG, 181.33 ± 4.27 mg/L) (Fig. 1c). In contrast, knocking down *ackA* did not enhance propionate production (PH, 123.67 ± 4.08 mg/L), likely due to the limited bacterial utilization of AA in the presence of glucose. However, as glucose levels decreased in the later stages of fermentation, the bacteria began to metabolize AA(Huang et al. 2018). This suggests that the low levels of AA in PH impaired the bacterial glyoxylate cycle, reducing the efficiency of coenzyme transport in the acrylic acid pathway. Additionally, growth curve analysis showed no significant difference in OD_600_ values among the engineered strain PG, the original EcN strain (EcN), and the empty vector control strain (EcN transformed with the pCDFDuet-1 vector alone, EcN-pCDF) throughout the cultivation period. All three strains exhibited similar delayed, logarithmic, and stable growth phases, indicating that the introduction of exogenous genes did not adversely affect the growth of EcN (Fig. 1d).Furthermore, propionate production from the EcN-pCDF control was negligible (Fig. 1c), confirming that the observed propionate synthesis was due to the introduced pathway genes and not the vector backbone. Consequently, PG was selected as the final strain for further analysis.

### 3.2 propionate production by engineered bacteria under different fermentation conditions

To further validate the propionate production of the engineered bacteria under different conditions, we conducted shake flask fermentations using *E. coli* universal complete medium M9, incomplete medium LB, and nutrient-rich YCFA medium. The results demonstrated that adding a certain proportion of glucose as a carbon source increased the propionate concentration in the fermentation broth (Fig. 1e). A comparison of fermentation results between incomplete medium LB and universal complete medium M9, both with the same glucose concentration, revealed that other carbon sources, such as proteins, could also influence propionate synthesis efficiency. Subsequently, shake flask fermentation of the engineered bacteria in LB medium supplemented with 10 g/L glucose resulted in the highest propionate production of 181.33 ± 4.27 mg/L by engineered bacteria PG (Fig. 1e).

### 3.3 addition of propionate-engineered probiotic PG can reduce abdominal irradiation-induced intestinal damage

**Fig. 2:**
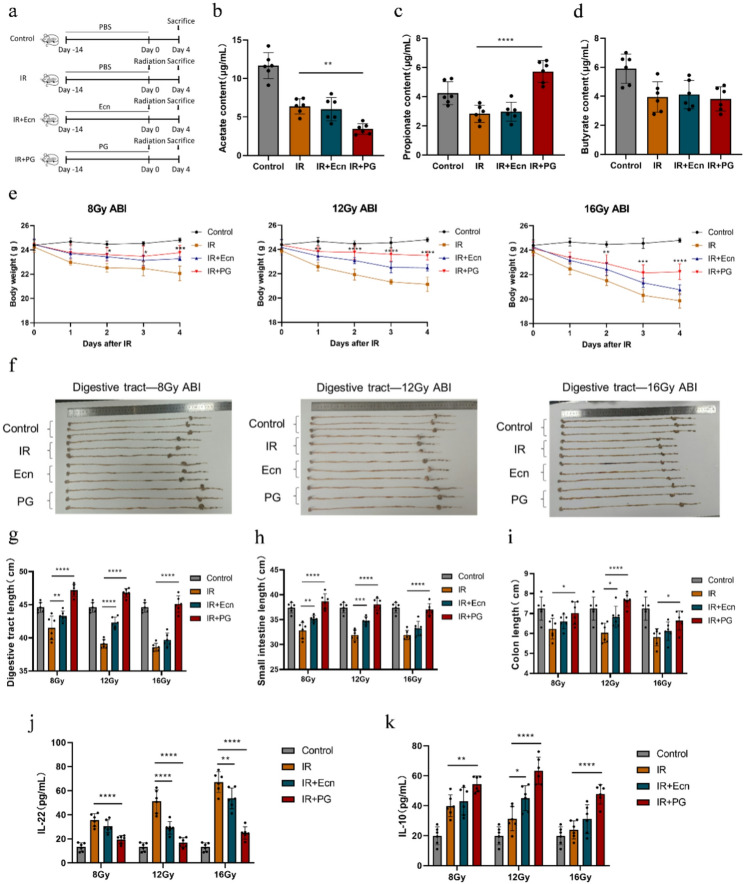
Supplementation with the propionate-engineered probiotic PG improved the overall physiological state of the body as well as that of the intestinal tract. **a** Scheme for probiotic EcN and engineered bacteria PG before ABI. Mice were euthanized on day 4 post-irradiation. **b**-**d** Acetate, propionate and butyrate content in intestinal contents (*n* = 6). **e** Changes in body weight of mice after abdominal irradiation(8 Gy, 12 Gy, 16 Gy) (*n* = 6). **f** Image of the digestive tract from three mice per group (8 Gy, 12 Gy, 16 Gy). **g** Digestive tract length at three irradiation doses(8 Gy, 12 Gy, 16 Gy) (*n* = 6). **h** Small intestine length at three irradiation doses(8 Gy, 12 Gy, 16 Gy) (*n* = 6). **i** Colon intestine length at three irradiation doses(8 Gy, 12 Gy, 16 Gy) (*n* = 6). **j** **k** The levels of IL-22 and IL-10 in intestine tissue at three irradiation doses(8 Gy, 12 Gy, 16 Gy) (*n* = 6). IL-22, Interleukin-22. Statistical analysis was conducted using one-way or two-way ANOVA. Error bars indicate standard errors, and values are expressed as the mean ± SD. *p < 0.05, **p < 0.01, ***p < 0.001, ****p < 0.0001; ns, no significance

Propionate is a key SCFAs in the intestinal tract, with a multifaceted and significant impact on intestinal health. Its effects are primarily seen in maintaining intestinal barrier function, regulating the microbiota, and modulating inflammation and immune responses (Yao et al. 2022; Liu et al. 2023). Probiotics, as crucial regulators of intestinal microecology, support intestinal health by maintaining environmental balance and boosting immunity (Xia et al. 2024). Propionate-engineered probiotics combine the dual benefits of propionate and probiotics, demonstrating remarkable synergistic effects. These engineered probiotics hold significant potential for preventing and treating intestinal diseases and for enhancing intestinal function (Kang et al. 2024). This study aimed to assess the protective role of propionate-engineered probiotics against radiation-induced abdominal injury. A C57BL/6J mouse model was used, which was randomly divided into four main experimental groups: the control group, the radiation-only group (IR), the probiotic chassis intervention group (IR + Ecn), and the engineered probiotic intervention group (IR + PG). Except for the control group, the remaining three groups were further subdivided into three subgroups (8 Gy, 12 Gy, and 16 Gy) according to the radiation dose. All groups were euthanized on day 4 following the completion of the corresponding abdominal irradiation dose (Fig. 2a). To assess the functional properties of the engineered probiotic PG in producing and delivering propionate, we quantified the concentration of propionate in the intestinal contents of mice. The results showed that the propionate level in the intestines of mice in the IR group was decreased. However, the PG intervention elevated the propionate level, effectively reversing the deficiency of this essential intestinal metabolite(Fig. 2d). Additionally, we measured the levels of acetate and butyrate in the intestinal contents and found that the PG intervention did not increase their concentrations(Fig. 2b-c).Under abdominal irradiation with doses of 8 Gy, 12 Gy, and 16 Gy, the IR group showed a significant decrease in body weight, whereas the IR + Ecn and IR + PG groups mitigated the body weight loss to varying degrees. Notably, compared to the 8 Gy and 16 Gy doses, the IR + PG group showed a more pronounced alleviation of body weight loss under the 12 Gy abdominal irradiation (Fig. 2e). Morphological analysis of the digestive tract in mice revealed that mice in the IR group that received 8 Gy, 12 Gy, and 16 Gy of radiation exhibited significant atrophy of the digestive tract compared to the control group(Fig. 2f). The lengths of the overall digestive tract, small intestine, and colon were shortened. However, both the probiotic EcN and engineered probiotic PG demonstrated varying degrees of protective effects on the digestive tract, promoting the restoration of the lengths of all digestive tract sections(Fig. 2g-i). Among them, the IR + Ecn and IR + PG groups showed the most significant intestinal restoration at the 12 Gy radiation dose. Abdominal irradiation induces an inflammatory response in the mouse intestine. Following abdominal irradiation, the levels of IL-22 and IL-10 were increased and decreased, respectively, in the intestines of mice. In contrast, intervention with probiotic EcN and engineered probiotic PG effectively reversed these trends and reduced the intestinal inflammatory response(Fig. 2j-k). Similarly, EcN and PG demonstrated the most significant improvement in inflammatory response at the 12 Gy radiation dose compared to other dose groups. The above results indicated that, among the three radiation doses, the IR + Ecn and IR + PG groups at the 12 Gy radiation dose had the most significant effect in ameliorating intestinal tissue damage caused by abdominal radiation exposure. Therefore, the 12 Gy radiation dose was selected for subsequent experiments.

**Fig. 3:**
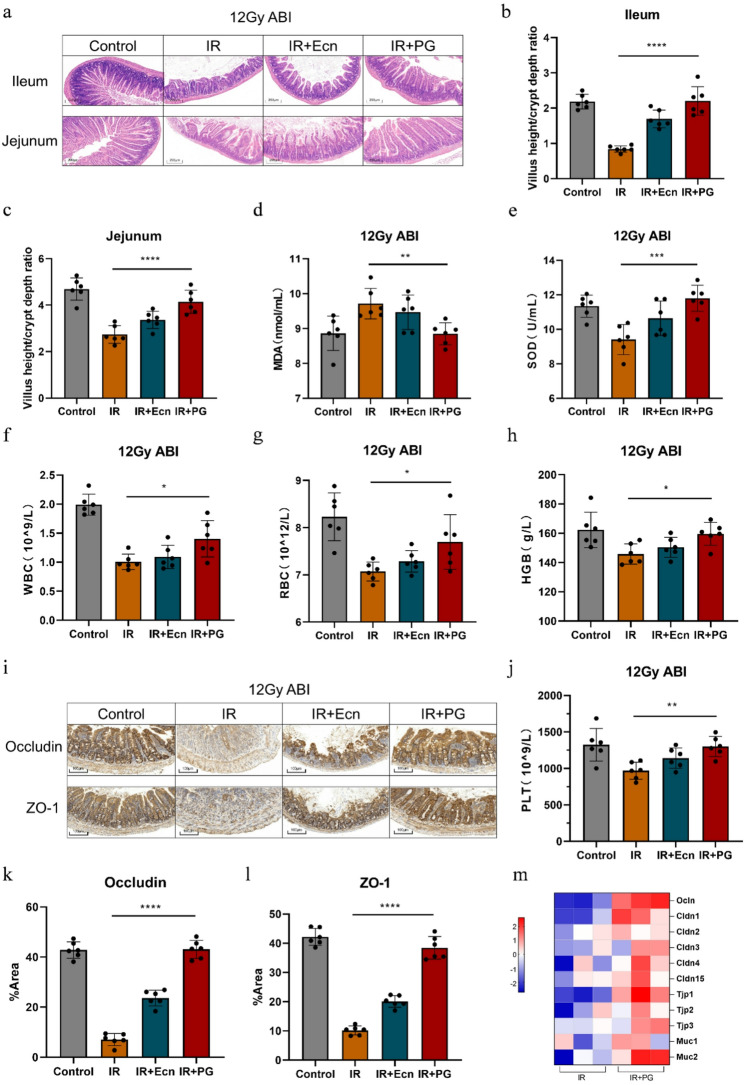
Supplementation with propionate-engineered probiotic PG reduced radiation-induced intestinal damage. **a** Ileum and jejunum tissues were stained with Hematoxylin-Eosin (HE) on day 4 post-radiation exposure(12 Gy). **b**, **c** The villus height/crypt depth ratio of ileum and Jejunum at 12 Gy irradiation doses (*n* = 6). **d** The level of malondialdehyde (MDA) in intestine tissue (*n* = 6). **e** The level of superoxide dismutase (SOD) in intestine tissue (*n* = 6). **f**-**h**, **j** The coment of white blood cells (WBC), red blood cells (RBC), hemoglobin (HGB) and blood platelet (PLT) (*n* = 6). (**i**, **k**-**l** Immunohistochemical images of Occludin and ZO-1 expression. **m** The heatmap showed the relative expression levels of tight junction protein-related genes in the IR and IR + PG groups. Statistical analysis was conducted using one-way ANOVA. Error bars indicate standard errors, and values are expressed as the mean ± SD. *p < 0.05, **p < 0.01, ***p < 0.001, ****p < 0.0001; ns, no significance

To assess the protective efficacy of the engineered probiotic PG against radiation-induced intestinal injuries, histopathological analyses were performed, focusing on the radiation-sensitive regions of the ileum and jejunum. The results of HE staining revealed that the IR group exhibited typical signs of radiation injury, including severe atrophy, thinning or even absence of villi, reduced crypt numbers, and inflammatory cell infiltration(Fig. 3a). The IR + Ecn group demonstrated moderate tissue repair, with villi elongation and partial restoration of crypt structures. In contrast, the IR + PG group showed superior structural preservation, characterized by more intact villus structures, normal crypt morphology, and markedly reduced inflammatory infiltration. Quantitative morphometric analysis corroborated these observations (Fig. 3b-c and Figure S2). Following intervention, both EcN and PG promoted the restoration of villus height and crypt depth across all radiation doses (8, 12, and 16 Gy). Importantly, the engineered probiotic PG exerted a more pronounced restorative effect than the probiotic chassis EcN. In the ileum, PG intervention increased the villus height/crypt depth (VH/CD) ratio by 57.80%, 161.60%, and 135.52% compared to the IR group at 8, 12, and 16 Gy, respectively. These improvements were substantially greater than those achieved by EcN (30.34%, 101.24%, and 19.18% at the corresponding doses). A similar trend was observed in the jejunum, where PG elevated the VH/CD ratio by 32.74%, 51.32%, and 41.64%, again outperforming the effects of EcN (21.22%, 22.86%, and 15.01%). Furthermore, the quantitative data indicated that the restorative efficacy of PG was most prominent at the 12 Gy abdominal irradiation dose, particularly in the ileum. These results collectively demonstrate that the propionate-engineered probiotic PG provides significant and dose-dependent histopathological protection against radiation-induced intestinal injury, with a superior therapeutic capacity compared to the probiotic chassis alone.

Abdominal irradiation induced oxidative stress in the organism (Wang et al. 2025), as evidenced by increased serum malondialdehyde (MDA) levels and reduced superoxide dismutase (SOD) levels (Fig. 3d-e). Both EcN and PG interventions effectively alleviated oxidative stress, with the PG group demonstrating a more pronounced reduction in MDA levels and a greater improvement in SOD levels. Abdominal radiation exposure can suppress myeloid hematopoiesis (Li et al. 2023), primarily leading to a significant decrease in peripheral blood cell parameters, including WBC, RBC, HGB, and PLT. The results indicated that the probiotic chassis EcN provided protection against radiation-induced bone marrow injury. Moreover, the genetically engineered PG strain demonstrated even greater efficacy, maintaining peripheral blood cell counts at physiological levels in the intervention group of mice (Fig. 3f-h and j). The thymus and spleen, as crucial immune organs, are particularly vulnerable to abdominal radiation. Studies have shown that abdominal irradiation can suppress thymic immunity, leading to tissue atrophy (Li et al. 2020). Simultaneously, it reduces spleen size and alters its cellular composition, thereby affecting blood filtration, circulation, and the body’s immune response. Experimental results confirmed that PG exerted a protective effect on these organs, preserving their structural and functional stability (Figure S3). The stable expression of tight junction proteins and mucins is essential for preserving intestinal barrier function and epithelial cell integrity (He et al. 2023). Immunohistochemical staining showed higher Occludin and ZO-1 expression in the PG-intervened group compared to the IR group, indicating enhanced tight junction integrity (Fig. 3i and k-l). Subsequently, we further analyzed the impact of PG on the expression of tight junction-related genes, and the results demonstrated that PG upregulated these gene expression levels (Fig. 3m). The above results indicated that both the probiotic EcN and the propionate-producing engineered probiotic PG alleviated intestinal tissue damage caused by abdominal radiation exposure and exerted a protective effect on irradiated mice. Notably, PG exhibited a superior protective effect compared to EcN, potentially due to its ability to continuously deliver propionate to the intestine.

**3.4 Engineered Probiotics PG can modulate the gut microbiota by enhancing the abundance of** ***Dubosiella*** **and** ***Akkermansia***.

**Fig. 4:**
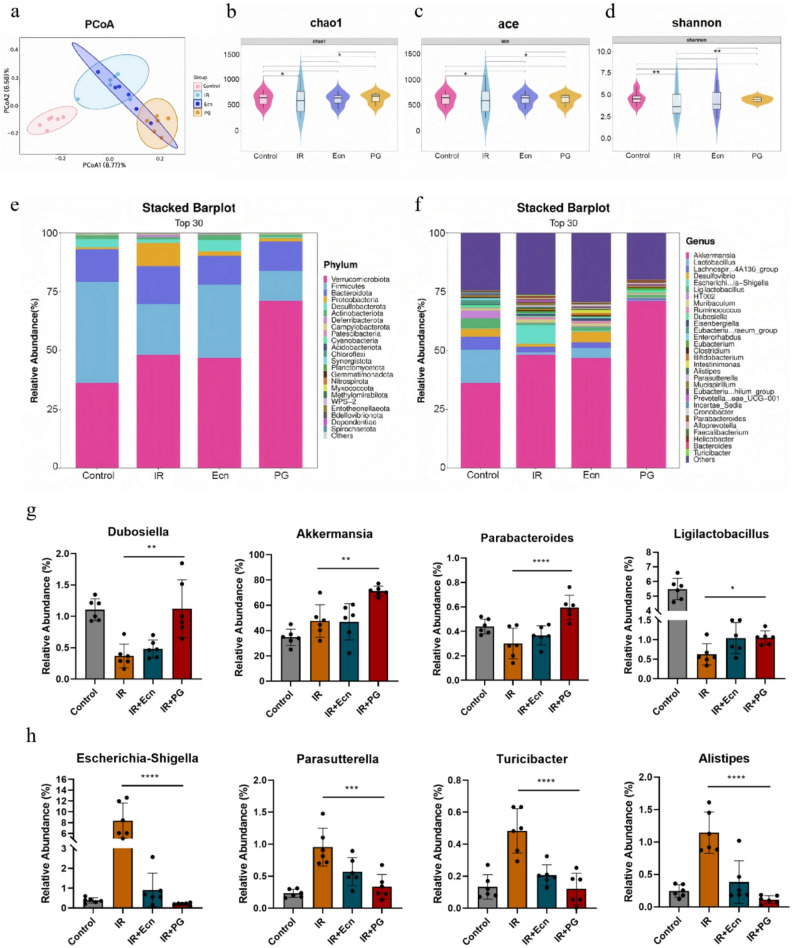
16 S rRNA gene sequencing analysis indicated that the engineered probiotic PG effectively protected the intestinal microbiota of irradiated mice. **a** Beta diversity analysis through Principal Coordinate Analysis (PCoA) revealed differences in microbiota composition across groups. **b**-**d** Alpha diversity violin plots, where the Chao1 and Ace indices represent microbial community richness, and the Shannon index reflects community diversity. **e**-**f**) Relative abundance of bacteria across different groups at the phylum and genus levels. **g** The relative abundances of intestinal probiotics, including *Dubosiella*, *Akkermansia*, *Parabacteroides*, and *Ligilactobacillus* (*n* = 6). **h** The relative abundances of harmful intestinal bacteria, including *Escherichia-Shigella*, *Parasutterella*, *Turicibacter*, and *Alistipes* (*n* = 6). Statistical analysis was conducted using one-way ANOVA. Error bars indicate standard errors, and values are expressed as the mean ± SD. *p < 0.05, **p < 0.01, ***p < 0.001, ****p < 0.0001; ns, no significance

Intestinal flora play a protective effect against radiation-induced intestinal damage through mechanisms such as maintaining barrier integrity, participating in metabolic processes, modulating immune responses, and promoting cell repair (Guo et al. 2020). In this study, we analyzed the regulatory effects of the engineered probiotic PG on the intestinal microbiota of mice subjected to 12 Gy abdominal irradiation, as well as its protective mechanism, using 16 S rRNA gene sequencing technology. The PCoA plot of beta diversity revealed a broader overall distribution of samples, with a distinct separation between the IR and PG groups. This suggests that the microbial community structures of these two groups exhibit different characteristics (Fig. 4a). Alpha diversity violin plots illustrate microbial community richness (based on the Chao1 and Ace indices) and community diversity (based on the Shannon index). Specifically, the richness and diversity of the microbiota in the IR group decreased following intraperitoneal irradiation. In contrast, PG effectively enhanced both the richness and diversity of the intestinal microbiota (Fig. 4b-d). The IR group exhibited a higher relative abundance of Proteobacteria at the phylum level compared to the control group, whereas EcN and PG interventions restored its abundance to levels comparable to the control group. Meanwhile, in the PG group, the abundance of *Verrucomicrobiota* reached its highest level (Fig. 4e), suggesting that EcN and PG exert a modulating effect on the post-irradiation intestinal microbiota. At the genus level, PG intervention increased the relative abundance of *Dubosiella* in the gut microbiota (Figs. 4f-g). *Dubosiella* has significant probiotic effects on the gut, where it ferments undigested carbohydrates to produce SCFAs, particularly propionic acid. Additionally, *Dubosiella* plays a vital role in regulating intestinal microecology. On one hand, it inhibits the growth of pathogenic bacteria including *Escherichia coli* and *Salmonella*; on the other hand, it promotes the proliferation of advantageous bacteria, optimizes the structure of the intestinal flora, and maintains the stability and harmony of the microbial community (Zhang et al. 2024a, b). Interestingly, the relative abundance of *Akkermansia* was higher in the PG group compared to the other three groups, which might be attributed to the propionic acid secreted by PG (Figs. 4f-g). *Akkermansia* is a beneficial bacterium present in the human intestinal tract, playing a key role in intestinal health by maintaining the intestinal barrier, modulating immune function, improving metabolic processes, and inhibiting the growth of pathogenic bacteria (He et al. 2023; Cani et al. 2022). EcN and PG interventions boosted the relative abundance of beneficial genera in the gut microbiota, including *Parabacteroides*, *Ligilactobacillus*, *Bacteroides*, *Bifidobacterium*, and *Faecalibacterium* (Fig. 4f-g and Figure S4a-c). *Parabacteroides* helps maintain intestinal flora balance by competitively inhibiting harmful bacteria and promoting the growth of advantageous bacteria. It also strengthens the intestinal barrier, regulates immune function, and participates in nutrient metabolism, exerting a significant positive impact on intestinal health (Cui et al. 2022). *Ligilactobacillus* (a taxon of the genus *Lactobacillus*) is a probiotic bacterium that plays a vital role in promoting digestion, maintaining intestinal homeostasis, and enhancing immune modulation (Wang et al. 2022). *Bacteroides* provide an energy source for the body and contribute to the construction of intestinal microecology, ensuring a stable gut environment (Wesener et al. 2024). *Bifidobacterium* is a well-known genus of beneficial bacteria that inhibits the growth of harmful bacteria, regulates intestinal immunity, and enhances nutrient absorption by producing acids. It plays a vital protective role in intestinal health across all age groups, including infants and adults (Henrick et al. 2021). As a dominant bacterial genus in the intestine, *Faecalibacterium* primarily supplies energy to intestinal epithelial cells by producing short-chain fatty acids (Yang et al. 2024a, b).

Meanwhile, radiation-induced disruption of the intestinal microenvironment promoted the proliferation of certain harmful bacteria. However, EcN and PG interventions suppressed the increased relative abundance of these genera (Fig. 4f, h and Figure S4d-e). *Escherichia-Shigella* includes pathogenic *E.coli* and *Shigella*, which can cause intestinal inflammation and compromise the intestinal mucosal barrier by adhering to and invading intestinal epithelial cells, as well as releasing toxins. This leads to symptoms such as diarrhea and abdominal pain, posing significant risks to intestinal health (Jaeggi et al. 2015). *Parasutterella*, a genus within the *Proteobacteria*, has been linked to intestinal inflammation in some studies, with its overproliferation potentially disrupting the intestinal mucosal barrier and triggering inflammatory responses (Henneke et al. 2022). *Turicibacter* may also be associated with adverse conditions, such as intestinal inflammation, and could exert harmful effects in certain cases (Kwon et al. 2023). Additionally, *Alistipes* has been suggested to exacerbate intestinal inflammation by modulating the function of intestinal immune cells and promoting the secretion of pro-inflammatory cytokines (Parker et al. 2020). Under conditions of intestinal dysbiosis, some harmful *Clostridium* species may overpopulate, disrupting the intestinal microecological balance and leading to intestinal dysfunction (Johanesen et al. 2015). *Cronobacter* may contribute to intestinal inflammation through the production of enterotoxins and other pathogenic factors (Song et al. 2023). The above results indicated that the engineered probiotic PG promoted the proliferation of advantageous bacteria while inhibiting the growth of pathogenic bacteria by increasing propionate levels, thereby regulating intestinal flora homeostasis.

### 3.5 engineered probiotic PG can modulate intestinal flora metabolism by influencing ascorbate and aldarate metabolism

**Fig. 5:**
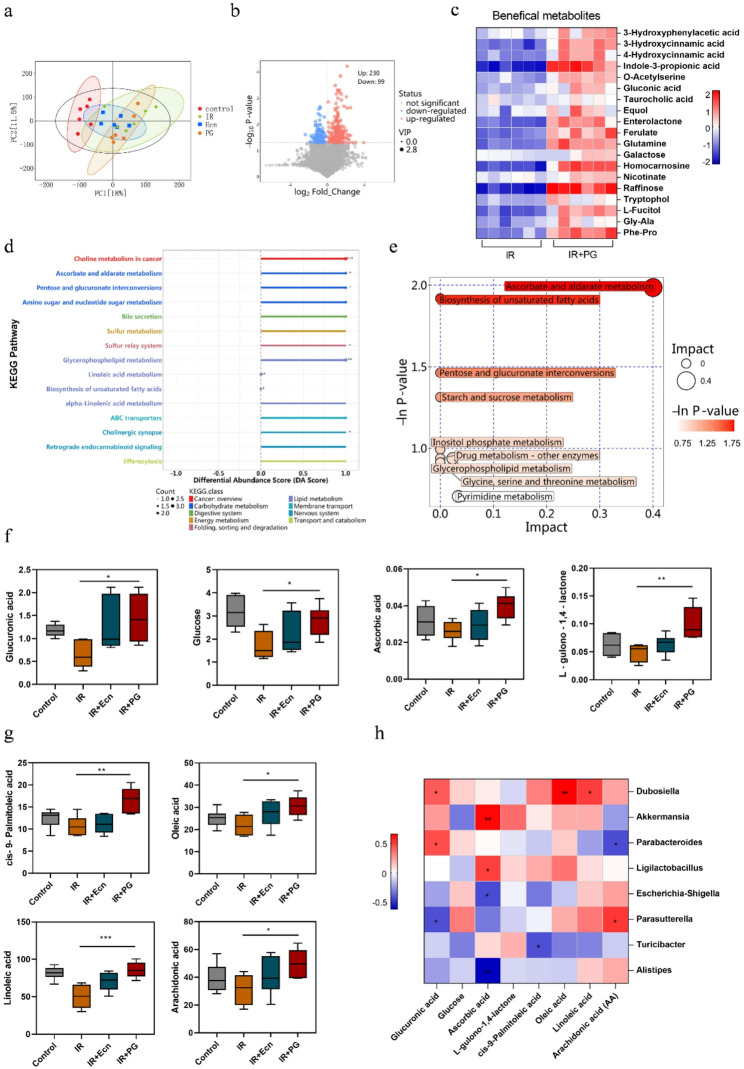
Metabolomics suggested that the engineered probiotic PG modulated the metabolism of the gut microbiota. **a** Principal Component Analysis (PCA) revealed differences in metabolic composition across groups. **b** The volcano plot illustrated the distribution of differential metabolites between the IR and IR + PG groups. **c** Heatmap analysis of beneficial metabolites showed the distribution of these metabolites between the IR and IR + PG groups. **d** Differential abundance scores were plotted by calculating the difference between upregulated and downregulated metabolites in key KEGG pathways, expressed as a proportion of the total metabolites in the pathway, to reflect overall changes in key metabolic pathways between the IR and IR + PG groups. **e** The results of metabolic pathway analyses are presented as bubble plots. The horizontal position and size of the bubbles represent the magnitude of the pathway’s influence factor in the topological analysis. The vertical position and color of the bubbles indicate the P-value of the enrichment analysis. **f** The boxplot displayed the relative levels of key metabolites involved in ascorbate and aldarate metabolism between the IR and IR + PG groups, including glucuronic acid, glucose, ascorbic acid, and L-gulono-1,4-lactone (*n* = 6). **g** The boxplot displayed the relative levels of key metabolites involved in biosynthesis of unsaturated fatty acids between the IR and IR + PG groups, including cis-9-Palmitoleic acid, oleic acid, linoleic acid and arachidonic acid (*n* = 6). **h** Heatmap illustrated the association between microbial communities and metabolites. Statistical analysis was conducted using one-way ANOVA. Error bars indicate standard errors, and values are expressed as the mean ± SD. *p < 0.05, **p < 0.01, ***p < 0.001, ****p < 0.0001; ns, no significance

Genetically engineered propionate-producing probiotics generate and release propionate in the gut, thereby optimizing the intestinal microenvironment (Kang et al. 2024). Additionally, the presence of engineered bacteria triggers changes in synergistic metabolic interactions among intestinal flora. For instance, SCFAs produced by engineered bacteria can be further metabolized by other bacteria to generate secondary metabolites, which are beneficial to intestinal health (Wu et al. 2024). This process enhances the metabolic network of the previously relatively independent intestinal flora, making it more interconnected and further optimizing the overall metabolic function of the intestinal microbiota. By analyzing gut content metabolites through metabolomics, we uncovered interactions between gut microbial metabolism and host metabolism. Principal component analysis (PCA) demonstrated distinct differences in metabolites among treatment groups (Fig. 5a). The volcano plot analysis identified a total of 329 differential metabolites, with 230 upregulated and 99 downregulated in the comparison between the IR group and the IR + PG group (Fig. 5b). The heatmap analysis revealed that PG intervention increased the levels of several beneficial metabolites compared to the IR group, including 3-Hydroxyphenylacetic acid, 3-Hydroxycinnamic acid, 4-Hydroxycinnamic acid, indole-3-propionic acid, o-Acetylserine, gluconic acid, taurocholic acid, equol, enterolactone, ferulate, glutamine, galactose, homocarnosine, nicotinate, Raffinose, tryptophol, fagitol, gly-Ala, phe-pro (Fig. 5c). These beneficial metabolites contribute to intestinal health through multiple mechanisms, including maintaining intestinal barrier homeostasis, regulating immunity, modulating gut microbiota, promoting cell proliferation and repair, and participating in energy metabolism and the synthesis of bioactive substances (Li et al. 2021; Lee et al. 2024; Mao et al. 2024). Differential Abundance Score analysis indicated a higher abundance of differential metabolites in several metabolic pathways in the IR + PG group, suggesting activation of these pathways (Fig. 5d). These included Choline metabolism in cancer, Ascorbate and aldarate metabolism, Pentose and glucuronate interconversions, Amino sugar and nucleotide sugar metabolism, Bile secretion, Sulfur metabolism, Sulfur relay system, Glycerophospholipid metabolism, Linoleic acid metabolism, Biosynthesis of unsaturated fatty acids, alpha-Linolenic acid metabolism, ABC transporters, Cholinergic synapse, Retrograde endocannabinoid signaling, and Efferocytosis.

To further identify differential metabolite pathways, we used bubble plots to highlight the pathways most associated with metabolite differences. The results revealed that ascorbate and aldarate metabolism were the most highly enriched pathways, followed by biosynthesis of unsaturated fatty acids (Fig. 5e). This suggests that the engineered probiotic PG may optimize the metabolic profile of gut microbes primarily by modulating these two metabolic pathways. Next, we used box plots to compare the levels of key metabolites. In ascorbate and aldarate metabolism, the key metabolites glucuronic acid, glucose, ascorbic acid and L - gulono-1,4-lactone were elevated in the IR + PG group (Fig. 5f). Ascorbate and aldarate metabolism plays a vital role in gut health. On one hand, ascorbate metabolism provides antioxidant protection for intestinal cells, shielding them from free radical damage and reducing the risk of intestinal inflammation (Traber et al. 2019). Additionally, this metabolic pathway promotes the proliferation and differentiation of intestinal epithelial cells, ensuring proper repair and renewal of the intestinal mucosal barrier. On the other hand, aldarate metabolism participates in the conversion of sugars and other substances in the intestinal tract, optimizing energy metabolism and regulating intestinal microecological balance, thereby indirectly supporting gut health (Simpson et al. 2024). Similarly, in biosynthesis of unsaturated fatty acids, the key metabolites cis-9-Palmitoleic acid, oleic acid, linoleic acid and arachidonic acid were elevated in the IR + PG group (Fig. 5g). Cis-9-Palmitoleic acid and oleic acid help maintain the fluidity and integrity of intestinal cell membranes, ensuring normal material exchange and signal transmission in intestinal cells (Bermúdez et al. 2022). Linoleic acid not only contributes to cell structure but also produces metabolic derivatives with anti-inflammatory properties that effectively suppress intestinal inflammation and reduce tissue damage (Jia et al. 2024). Arachidonic acid plays a vital role in regulating intestinal immunity and precisely modulating immune cell activity. Additionally, it influences the growth environment and nutrient supply of intestinal flora, supporting the proliferation of beneficial bacteria while inhibiting the expansion of harmful bacteria, thereby synergistically maintaining the stability and balance of the intestinal microecology (Xu et al. 2023). The above results indicated that the propionate-engineered probiotic PG primarily regulates metabolism through ascorbate and aldarate metabolism, as well as biosynthesis of unsaturated fatty acids. The integration of 16 S rRNA sequencing and metabolomics data revealed strong positive correlations between *Dubosiella* and oleic acid, as well as *Akkermansia* and ascorbic acid. Based on these findings, we speculate that PG may regulate ascorbate and aldarate metabolism, along with biosynthesis of unsaturated fatty acids, by increasing the relative abundance of *Dubosiella* and *Akkermansia*. This mechanism may contribute to alleviating radiation-induced intestinal damage.

### 3.6 Engineered probiotic PG protects the gut from radiation-induced damage, potentially via modulation of the SOCS1/JAK2/STAT3 axis

**Fig. 6:**
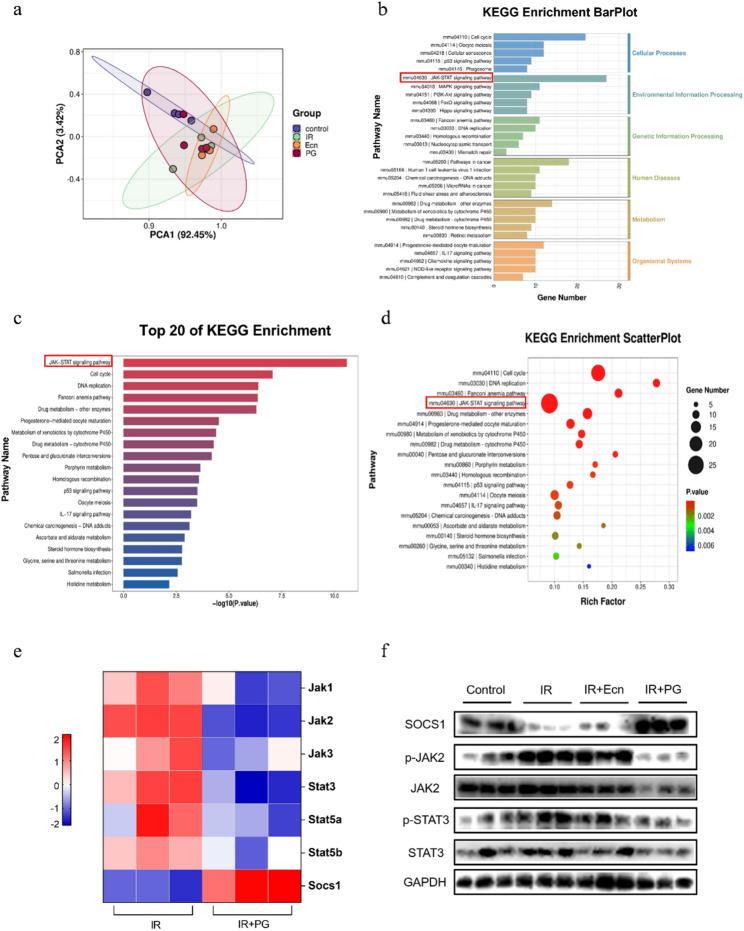
Engineered probiotic PG mitigated radiation-induced intestinal injury by regulating the SOCS1/JAK2/STAT3 pathway. **a** Principal Component Analysis (PCA) revealed differences in gene expression levels among groups. **b** KEGG Enrichment BarPlot for IR vs. IR + PG displayed the number of differential genes in the pathway represented on the horizontal axis, pathway names on the vertical axis, and colors representing KEGG Level 1 classification. **c** KEGG-enriched bar graph displaying the top 20 KEGG pathways with the smallest P-values. **d** The results of the KEGG enrichment analysis werw presented as a scatter plot. (e) The heatmap illustrated the relative expression levels of JAK-STAT signaling pathway-related genes in the IR and IR + PG groups. **f** Western blot analysis demonstrated the protein expression levels of SOCS1, p-JAK2, JAK2, p-STAT3, and STAT3 across groups

To investigate the molecular mechanism by which engineered probiotic PG alleviates radiation-induced intestinal injury, we performed RNA transcriptome sequencing of intestinal tissues from mice in the PG intervention group. Principal component analysis revealed that the gene expression profiles of the IR + Ecn and IR + PG groups were distinct from those of the IR group, indicating that EcN and PG interventions altered intestinal transcriptional regulation patterns (Fig. 6a). Next, the differentially expressed genes (DEGs) were systematically analyzed for functional enrichment. Using the Kyoto Encyclopedia of Genes and Genomes (KEGG) metabolic pathway database, these DEGs were mapped to their corresponding metabolic pathways, and enriched pathways were identified. KEGG Enrichment BarPlot revealed that the DEGs were primarily associated with Environmental Information Processing and Cellular Processes (Fig. 6b). Notably, the most enriched pathway within Environmental Information Processing was the JAK-STAT signaling pathway (Fig. 6c). Similarly, KEGG Enrichment ScatterPlot confirmed that the JAK-STAT signaling pathway was the most enriched pathway (Fig. 6d). The crucial role of the JAK-STAT signaling pathway in maintaining inflammatory homeostasis suggests its potential involvement in mitigating radiation-induced intestinal injury in mice. Heatmap analysis revealed that several key genes within the JAK-STAT signaling pathway were downregulated following PG intervention, including *Jak1*, *Jak2*, *Jak3*, *Stat3*, *Stat5a*, and *Stat5b*, whereas *Socs1* exhibited high expression (Fig. 6e). Among them, changes in *Jak2*, *Stat3*, and *Socs1* genes were the most significant. Inhibition of the JAK-STAT signaling pathway reduces intestinal inflammatory responses and mitigates tissue damage caused by excessive immune cell activation, thereby helping to maintain intestinal immune homeostasis (Wang et al. 2024a, b; Chen et al. 2024a, b). SOCS1 is a key negative feedback regulator of the JAK-STAT pathway. It binds to activated JAK kinases, inhibiting their activity and preventing further phosphorylation of STAT proteins, thereby terminating JAK-STAT signal transduction. Through this negative feedback mechanism, SOCS1 prevents excessive signal activation and maintains immune balance (Liau et al. 2018; Philips et al. 2022). Western blot results further confirmed that the protein levels of p-JAK2 and p-STAT3 were higher in the IR group than in the Control group, while SOCS1 protein expression was reduced. In contrast, PG intervention effectively downregulated p-JAK2 and p-STAT3 protein expression while upregulating SOCS1 protein expression (Fig. 6f). The activation state of the JAK-STAT pathway, a key signaling pathway associated with stress-induced inflammation, can be characterized by the expression levels of phosphorylated JAK and STAT proteins. These results suggest that the propionate-engineered probiotic PG mitigates radiation-induced intestinal damage, a protective effect associated with the modulation of the SOCS1/JAK2/STAT3 axis and inhibition of the JAK-STAT signaling pathway.

## 4. Discussion

In this study, an engineered probiotic strain capable of stable propionate production was successfully constructed using synthetic biology. Its performance was enhanced by optimizing gene expression and knocking out genes associated with bypass metabolic pathways. These modifications led to improved propionate production, and the engineered PG strain was identified as having excellent performance. Furthermore, the introduction of exogenous genes did not affect its growth, and the strain demonstrated good stability. Next, the mice were exposed to three different radiation doses: 8 Gy, 12 Gy, and 16 Gy to induce intestinal damage. The engineered probiotic strain was then administered as a treatment for radiation-induced intestinal injury. Our findings showed that the engineered strain stably and efficiently released propionate in the intestinal tract, thereby mitigating radiation-induced damage. Notably, the therapeutic effect of the engineered bacteria was most pronounced at a radiation dose of 12 Gy. Meanwhile, the engineered strain regulated intestinal flora homeostasis by enhancing the abundance of advantageous bacteria, including *Dubosiella* and *Akkermansia*. Additionally, it modulated intestinal flora metabolism by influencing key metabolic pathways, including ascorbate and aldarate metabolism, thereby enhancing anti-inflammatory responses and strengthening barrier protection functions. Further studies revealed that the protective effect of PG was associated with the modulation of the SOCS1/JAK2/STAT3 signaling axis, a key pathway implicated in inflammatory homeostasis, potentially contributing to the mitigation of radiation-induced intestinal damage.

In modern oncology, radiotherapy stands as a powerful weapon against malignant tumors, offering countless patients a renewed hope for survival (Aboagye et al. 2023). However, radiation-induced bowel injury remains a major complication, often leading to debilitating side effects including diarrhea and intestinal fistula, which impact patients’ quality of life. Thus, identifying effective strategies to alleviate the adverse effects of radiotherapy is of great clinical significance. While previous studies have primarily focused on the enteroprotective effects of butyrate (Yang et al. 2024a, b; Salvi and Cowles 2021), emerging evidence suggests that, compared to acetate and butyrate, propionate exhibits a more pronounced radioprotective effect (Guo et al. 2020; He et al. 2023). As a substance with significant therapeutic potential, propionate plays a vital role in maintaining intestinal homeostasis and modulating immune function (Wang et al. 2024a, b; Filippone et al. 2020). Known mechanisms include binding to G-protein-coupled receptors (e.g., GPR43) on immune and epithelial cells, and acting as a histone deacetylase (HDAC) inhibitor, both of which can dampen pro-inflammatory signaling (Bilotta et al. 2021; Cong et al. 2022; Adesina et al. 2021).However, direct supplementation with propionate is hindered by its low bioavailability and limited sustained effect (Shin et al. 2023), making widespread clinical application challenging. To address these limitations, we constructed an engineered probiotic strain capable of producing propionate. EcN was selected as the chassis due to its compatibility with synthetic biology tools and its well-established safety record (Yu et al. 2023). By genetically engineering this probiotic to produce propionate, we not only overcame the shortcomings of direct supplementation but also leveraged the colonization and replication properties of probiotics to ensure precise and sustained delivery of propionate to the intestinal tract. This approach enhances the therapeutic potential of propionate by providing continuous local supplementation in a targeted and safe manner, thereby maximizing its gut health-promoting effects. Probiotics offer multiple benefits, including protecting the intestinal barrier, maintaining intestinal homeostasis, and enhancing immune function (Chandrasekaran et al. 2024). When combined with propionate, they produce a synergistic effect, further amplifying therapeutic efficacy. In this study, the dual strategy of propionate delivery and probiotic colonization addressed key limitations of existing radiation enteritis treatments, such as the low bioavailability of direct propionate supplementation and the insufficient targeting of traditional probiotics. Compared with synthetic drugs like octreotide and amphotericin, the engineered probiotic strain enables the continuous release of propionate within the intestine, thereby minimizing systemic side effects. This innovative approach not only effectively mitigates radiation-induced intestinal damage but also introduces a novel therapeutic avenue for radiotherapy.

This study also revealed that engineered probiotics have significant effects on intestinal flora and metabolism. The engineered bacteria regulated the composition of the intestinal microbiota, increasing both its abundance and diversity while promoting the growth of advantageous bacteria including *Dubosiella* and *Akkermansia*. Numerous studies have demonstrated that these advantageous bacteria are essential for maintaining intestinal health (Zhang et al. 2024a, b; Cani et al. 2022; Cui et al. 2022; Wang et al. 2022; Wesener et al. 2024; Henrick et al. 2021; Yang et al. 2024a, b). He et al. reported that *Akkermansia muciniphila* alleviates radiation-induced intestinal damage by secreting propionate (He et al. 2023). In this study, propionate-producing engineered bacteria were found to promote the proliferation of *Akkermansia*, suggesting that increased propionate production could further enhance the intestinal barrier. Together, they form a synergistic positive feedback loop that helps maintain intestinal microecological balance. Metabolically, the engineered bacteria regulated intestinal flora metabolism by activating pathways such as ascorbate and aldarate metabolism and biosynthesis of unsaturated fatty acids, producing a variety of metabolites beneficial to intestinal health. These metabolites contribute to intestinal health in multiple ways, including maintaining barrier function, modulating immunity, regulating microbiota composition, promoting cell proliferation and repair, and participating in energy metabolism (Traber et al. 2019; Simpson et al. 2024; Bermúdez et al. 2022; Jia et al. 2024; Xu et al. 2023).

The immunomodulatory properties of propionate are mediated through diverse signaling pathways. In immune cells, including macrophages and dendritic cells, propionate has been shown to suppress pro-inflammatory responses by inhibiting NF-κB activation and NLRP3 inflammasome activity, thereby promoting an anti-inflammatory phenotype (Fock and Parnova 2023; Zhuang et al. 2023). Concurrently, in intestinal epithelial cells, propionate enhances barrier integrity and repair processes through mechanisms involving AMPK and HIF-1α signaling (Jiang et al. 2025; Yao et al. 2025; Liu et al. 2025). Furthermore, evidence indicates that SCFAs, including propionate, can modulate the JAK-STAT pathway, a central coordinator of cytokine-driven immune responses (Bilotta et al. 2021; Zhao et al. 2024). Our findings extend this understanding to the context of radiation-induced gastrointestinal injury. Specifically, the sustained intestinal delivery of propionate via the engineered probiotic PG was associated with a distinct molecular signature: upregulation of the negative feedback regulator SOCS1 accompanied by reduced phosphorylation of JAK2 and STAT3. This pattern aligns with the established capacity of propionate to fine-tune inflammatory signaling by reinforcing endogenous negative regulatory circuits. While the precise upstream molecular initiators of this effect warrant further investigation, our in vivo data substantiate a link between microbiota-derived propionate and attenuation of the JAK-STAT/SOCS1 axis within the complex pathophysiology of radiation enteropathy.

Building upon the established immunomodulatory framework of SCFAs, our mechanistic data further indicate that the protective effect of the engineered bacteria correlated with the modulation of specific inflammatory signaling pathways. It has been demonstrated that inhibition of the JAK-STAT signaling pathway reduces intestinal inflammation, decreases tissue damage caused by the over-activation of immune cells, and helps maintain intestinal immune homeostasis (Salas et al. 2020; Shi et al. 2025). In the present study, the protection offered by propionate-producing engineered probiotics correlated with a signature of inhibition in the JAK-STAT signaling pathway, characterized by modulation of the SOCS1/JAK2/STAT3 axis. It should be noted that the present in vivo evidence demonstrates a strong correlation between PG intervention and modulation of the SOCS1/JAK2/STAT3 axis, rather than defining a precise causal mechanism. The exact means by which bacterially delivered propionate influences this host signaling pathway-potentially involving direct receptor interaction, epigenetic modification, or other intermediate processes-requires further elucidation. Consequently, dissecting the upstream mechanistic events through which propionate modulates this signaling axis constitutes an essential focus for future investigation to establish causality and advance the functional understanding of this protective interaction.

This study confirmed that propionate-producing engineered probiotics can effectively mitigate radiation-induced intestinal damage via a multidimensional action that involved microbial homeostasis restoration, metabolic reprogramming, and was associated with modulation of host inflammatory signaling. Moreover, its modular design can be adapted for the targeted delivery of other metabolites, such as cytokines and enzymes, offering new possibilities for the precise treatment of gastrointestinal diseases. Furthermore, while this study focused on acute barrier restoration in the radiation-sensitive small intestine, future investigations into the effects of sustained propionate delivery on colonic adaptation, including the expression and function of SCFA transporters and receptors in chronic injury models, will provide a more comprehensive understanding of its therapeutic scope. By integrating synthetic biology, metabolomics, and transcriptomics, this approach not only provides an innovative strategy for the clinical prevention and treatment of radiation enteritis but also highlights the potential of engineered probiotics in addressing complex gastrointestinal disorders. With continued research and clinical validation, this genetically engineered probiotic holds promise as a novel biotherapeutic for radiation-induced enterocolitis, ultimately improving patients’ quality of life.

## Supplementary Information

Below is the link to the electronic supplementary material.

Supplementary Material 1

Supplementary Material 2

Supplementary Material 3

## Data Availability

The 16 S rRNA gene sequencing and transcriptome sequencing raw data generated in this study have been submitted to the NCBI database under accession numbers PRJNA1237540 and PRJNA1237809. Additional data supporting the validation of the paper’s conclusions are provided in the main text and Supplementary Material sections.

## Declarations

In this study, we strictly complied with the ethical requirements for experimental animals and established clear humane endpoints. During the course of the experiments, some mice in the untreated and treated groups developed serious health problems, such as weight loss of more than 20%, blood in the stool, and locomotor disorders. To protect the welfare of the animals, we immediately terminated these experiments and euthanized them to alleviate their suffering. This practice demonstrated our respect for the lives of experimental animals while ensuring the scientific reliability of the experimental data.
