# Supplementary material for: Propionate-engineered probiotics reduce radiation-induced intestinal damage
Source: Bioresour Bioprocess. 2026 Feb 17;13(1):25. doi: 10.1186/s40643-026-01020-9 (PMC12913845; doi:10.1186/s40643-026-01020-9)

**The whole un-cropped images of the original western blots**

GAPDH and P-STAT3-Original Western Blots：


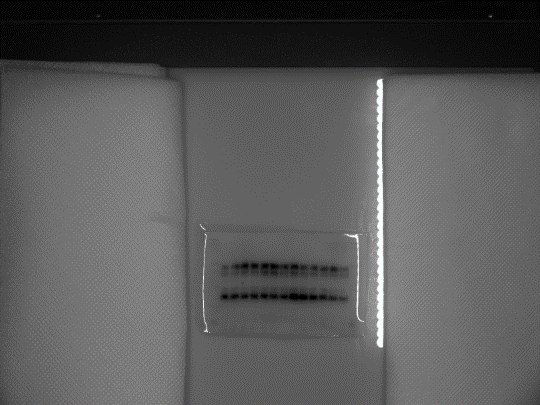


STAT3-Original Western Blots：


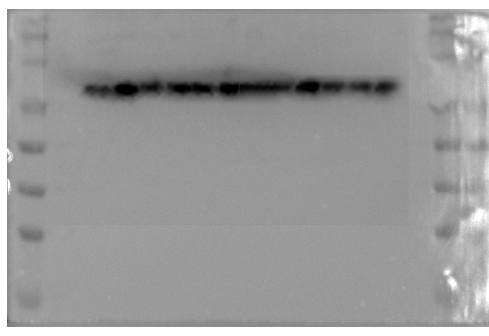


P-JAK2-Original Western Blots：


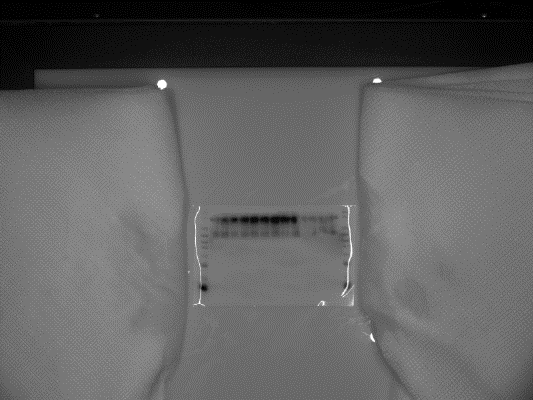


JAK2-Original Western Blots：


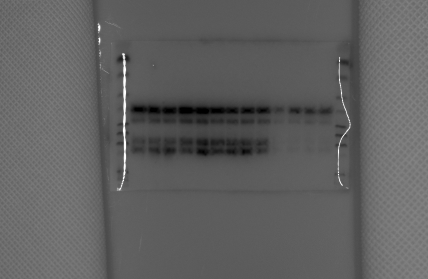


SOCS1-Original Western Blots：


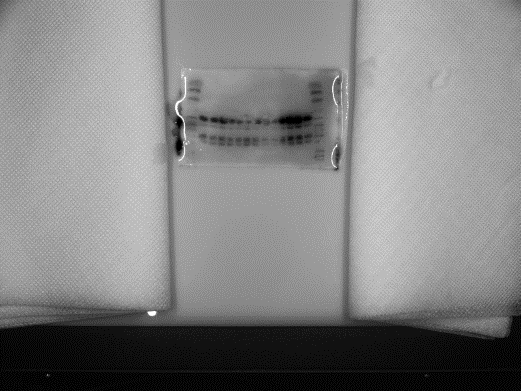

Supplement: Supplementary file 1 — Supplementary Material 1 [file 40643_2026_1020_MOESM1_ESM.docx]
