# Supplementary material for: Propionate-engineered probiotics reduce radiation-induced intestinal damage
Source: Bioresour Bioprocess. 2026 Feb 17;13(1):25. doi: 10.1186/s40643-026-01020-9 (PMC12913845; doi:10.1186/s40643-026-01020-9)
Supplement: Supplementary file 2 — Supplementary Material 2 [file 40643_2026_1020_MOESM2_ESM.docx]

**Supplementary materials**

**
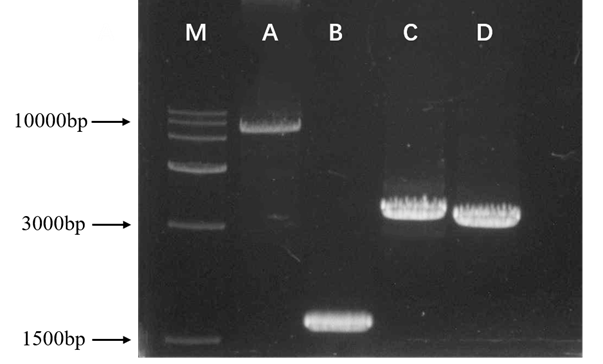
**

**Figure S1.** Electrophoretic validation of exogenous gene fragments in engineered bacteria

(M: Trans 15K DNA marker; A: pct-lcd-acr gene fragment; B: pct gene fragment;

C: lcd gene fragment; D: acr gene fragment).


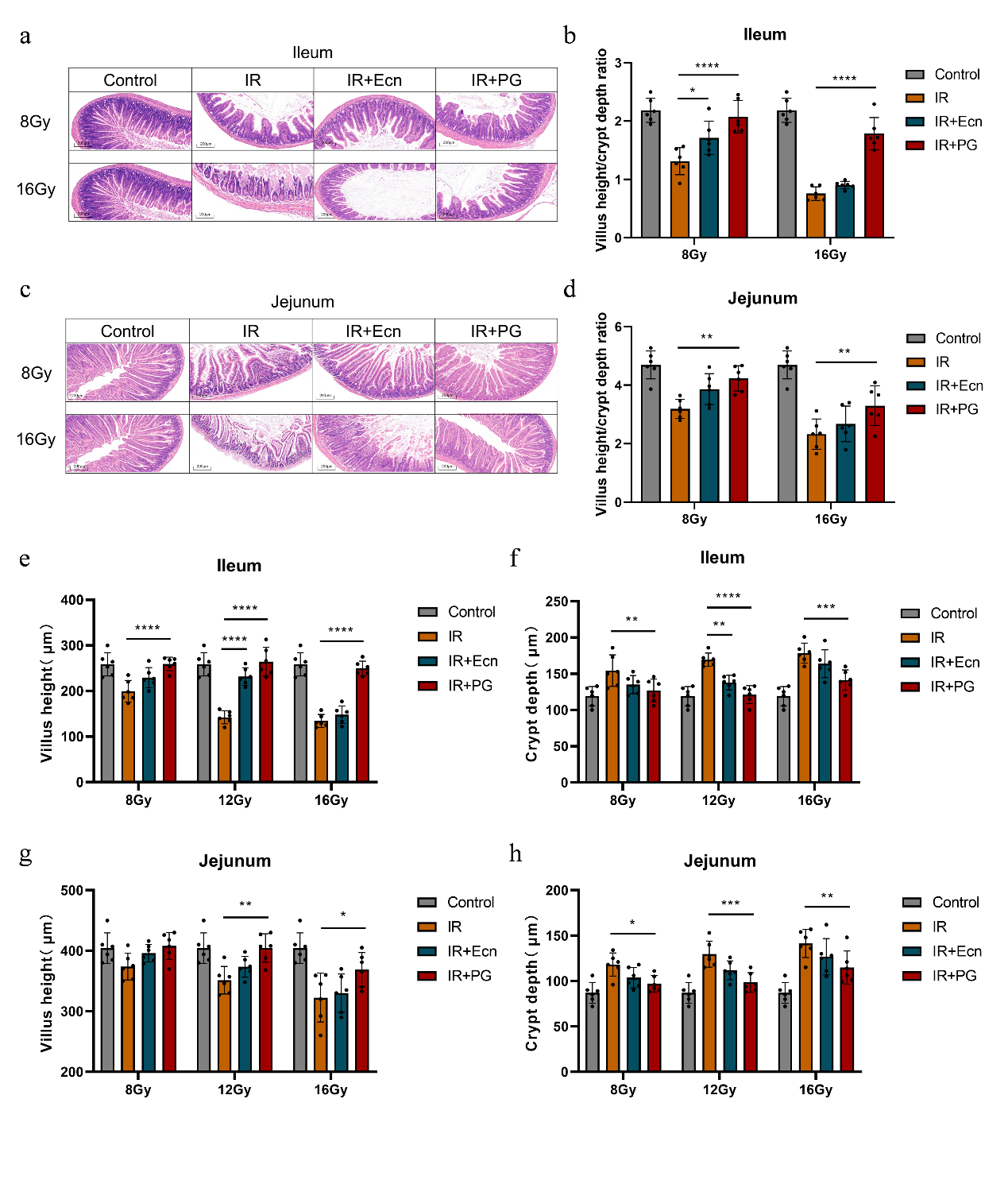


**Figure S2.** Histopathological analysis of the ileum and jejunum intestines. (a,c) Hematoxylin-Eosin (HE) staining results of the ileum and jejunum at 8 Gy and 16 Gy radiation doses. (b,d) The villus height/crypt depth ratio of ileum and Jejunum at 8Gy and 16 Gy irradiation doses (n=6). (e-h) Villus length and crypt depth in the ileum and jejunum at three radiation doses(8Gy, 12Gy, 16Gy) (n=6).


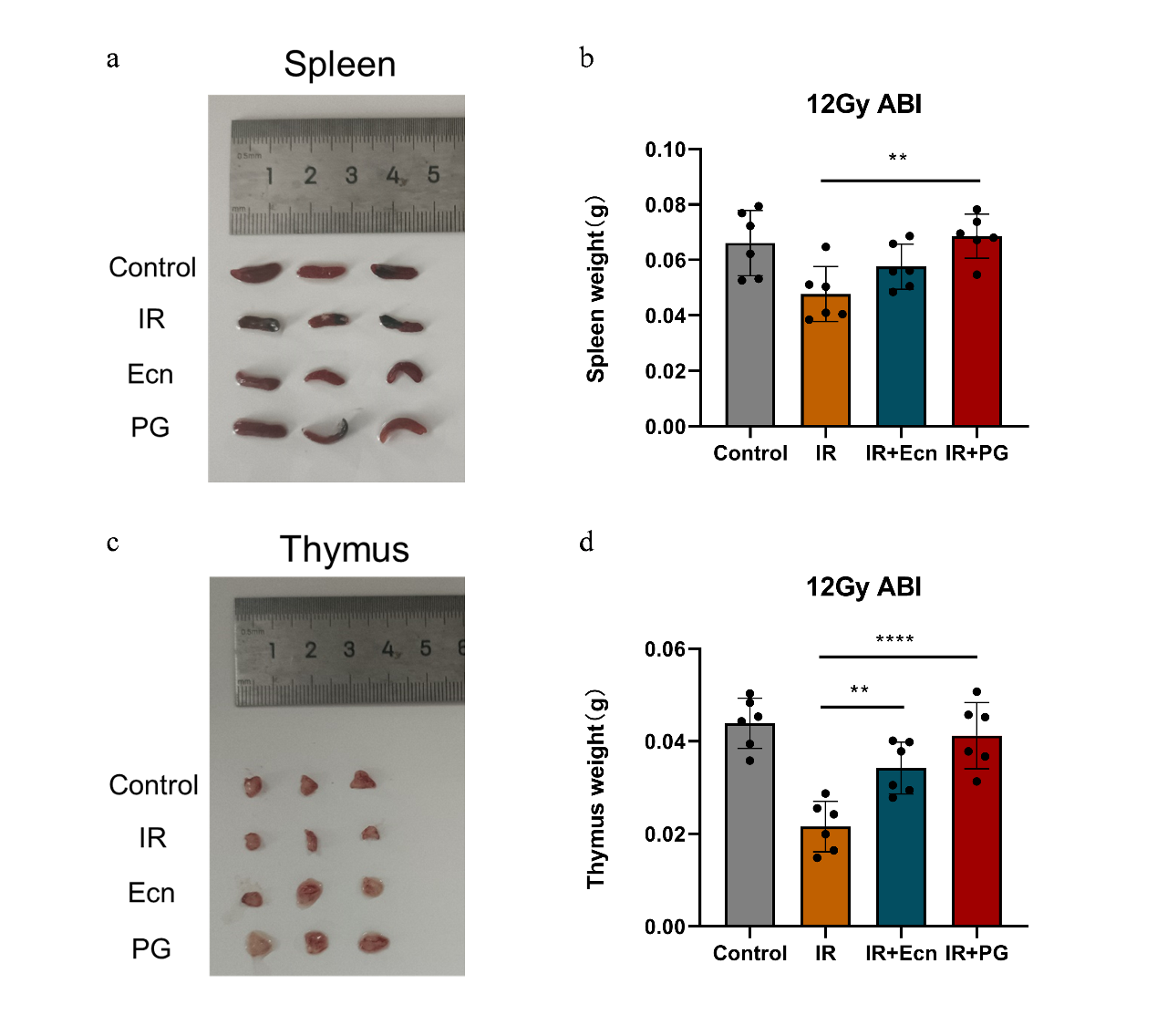


**Figure S3.** Representative images and weights of the spleen and thymus in each group of mice. (a) Spleen image of three mice from each group. (b) Spleen weight of mice(n=6). (c) Thymus image of three mice from each group. (d) Thymus weight of mice(n=6).


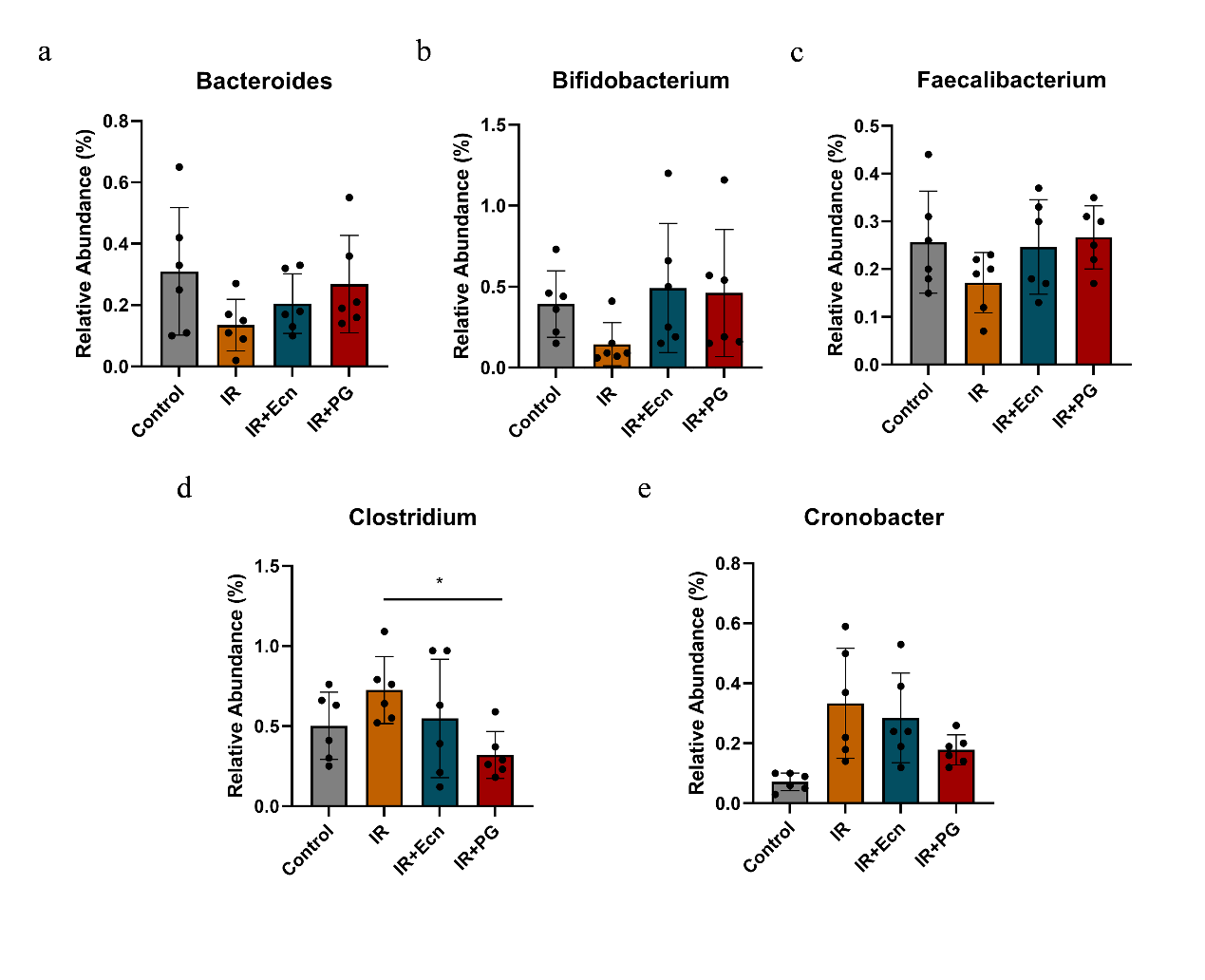


**Figure S4.** The relative abundance of beneficial and harmful gut bacteria. (a-c) The relative abundance of beneficial gut bacteria, including *Bacteroides*, *Bifidobacterium*, *Faecalibacterium*. (d,e) The relative abundance of harmful gut bacteria, including *Clostridium*, *Cronobacter*.

**Table S1.** Strains and plasmids used in this study

| Strain or plasmid | Relevant properties | Source |
| --- | --- | --- |
| *E. coli* DH5α | F^–^ φ80*lac*ZΔM15Δ(*lac*ZYA-*arg*F)U169 *end*A1*rec*A1 | TransGen  Biotech |
| *E. coli* Nissle 1917 | wild type | Lab stock |
| PE | *E. coli* Nissle1917 harboring pCDFDuet-*pct*-*lcd*-*acr* | This study |
| PF | *E. coli* Nissle1917 harboring pCDFDuet-*pct*-*lcd*-*acr*, *ΔpflB* | This study |
| PG | *E. coli* Nissle1917 harboring pCDFDuet-*pct*-*lcd*-*acr;* *ΔpflB; ΔpoxB* | This study |
| PH | *E. coli* Nissle1917 harboring pCDFDuet-*pct*-*lcd*-*acr;* *ΔpflB; ΔpoxB; ΔackA* | This study |
| pCDFDuet | CloDF13 replicon; streptomycin | Lab stock |
| pCDFDuet-*pct*-*lcd*-*acr* | CloDF13 replicon; *pct; lcd; acr*; streptomycin | This study |
| pKD46 | pSC101 replicon; ampicillin | Lab stock |
| pKD3 | R6K γ replicon; chloramphenicol | Lab stock |
| pCP20 | pSC101 replicon; ampicillin; chloramphenicol | Lab stock |
