# Supplementary figures and images for: Propionate-engineered probiotics reduce radiation-induced intestinal damage
Source: Bioresour Bioprocess. 2026 Feb 17;13(1):25. doi: 10.1186/s40643-026-01020-9 (PMC12913845; doi:10.1186/s40643-026-01020-9)

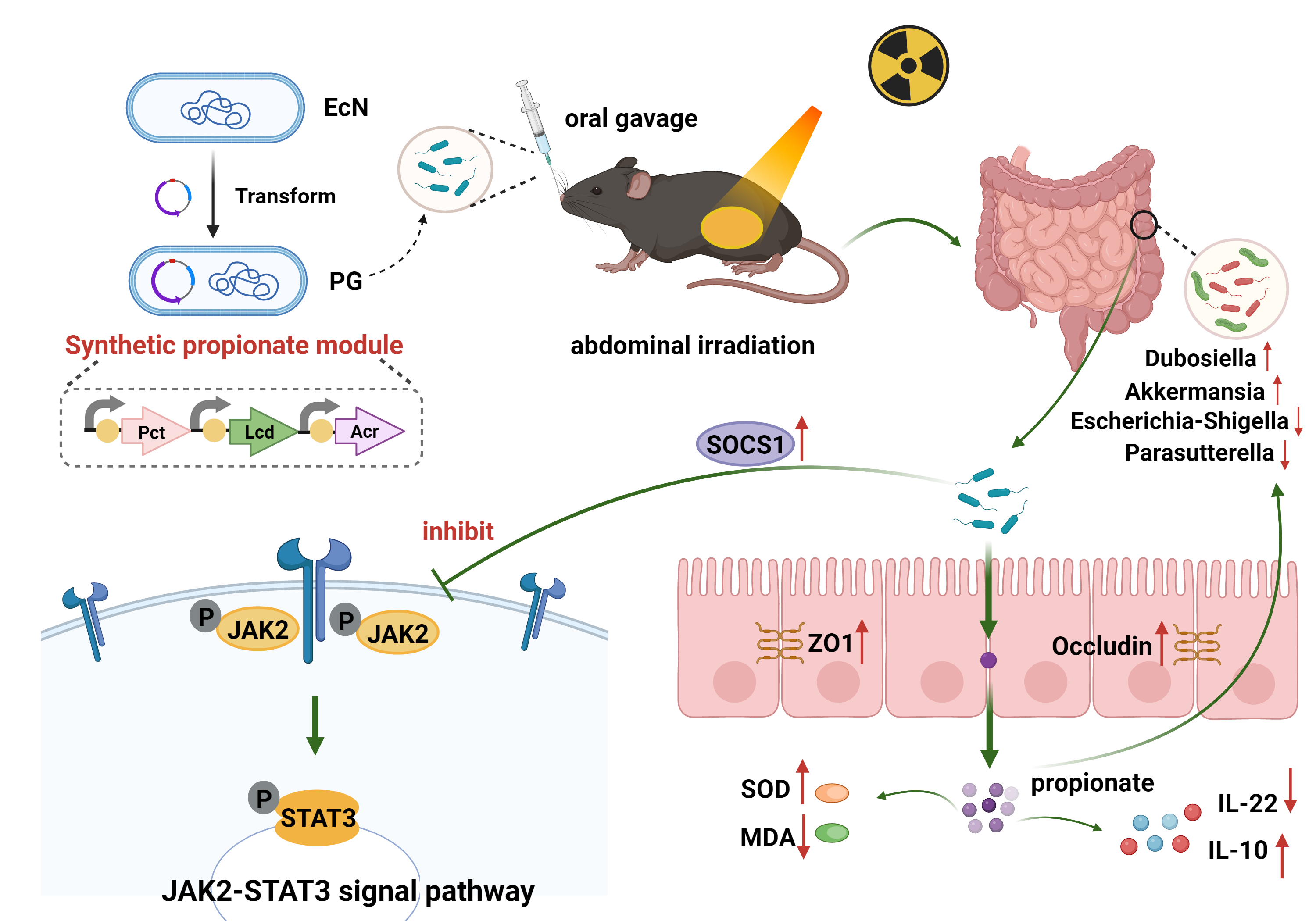

Supplement: Supplementary file 3 — Supplementary Material 3 [file 40643_2026_1020_MOESM3_ESM.png]
